# Efficacy and Safety of Generic Fluocinolone Acetonide, Hydroquinone, and Tretinoin Cream Compared With TRI‐LUMA for the Treatment of Moderate‐To‐Severe Melasma in Chinese Patients: A Randomized, Single‐Center, Placebo‐Controlled Trial

**DOI:** 10.1111/jocd.70205

**Published:** 2025-04-28

**Authors:** Honghua Hu, Pengfei Zhou, Hongliang Yao, Chengyao Zhu, Bo Shen, Bo Feng, Xiangke Yu, Lunfei Liu

**Affiliations:** ^1^ Department of Dermatology The Fourth Affiliated Hospital of School of Medicine, and International School of Medicine, International Institutes of Medicine, Zhejiang University Yiwu China; ^2^ Department of Dermatology Linping Hospital of Integrated Traditional Chinese and Western Medicine Hangzhou China

**Keywords:** efficacy, fluocinolone acetonide, hydroquinone, and tretinoin cream, melasma, safety

## Abstract

**Background:**

Treatment with fluocinolone acetonide, hydroquinone, and tretinoin cream is the gold standard for melasma; however, the effects of this treatment in the Chinese population remain unclear. Due to the differences between Chinese and Caucasian subjects, further clinical trials in Chinese patients with melasma are needed.

**Aim:**

To evaluate the efficacy and safety of fluocinolone acetonide, hydroquinone, and tretinoin creams in Chinese patients with moderate‐to‐severe melasma.

**Methods:**

We recruited 53 patients who received a generic formulation (triple combination cream, TCC), brand formulation (TRI‐LUMA), or placebo once daily for 8 weeks. In weeks 4 and 8, efficacy was evaluated based on the Melasma Severity Scale and Melasma Area and Severity Index. In addition, safety was assessed based on the incidence and severity of treatment‐emergent adverse events.

**Results:**

The generic TCC group achieved 52.2% efficacy compared to 57.1% in the TRI‐LUMA group. There was no significant difference in the incidence of adverse effects between the TCC and TRI‐LUMA groups (69.6% and 90.5%, respectively; *p* > 0.05). The incidence of adverse events in the placebo group was 0%.

**Conclusion:**

Generic TCC was as effective as TRI‐LUMA and had a similar safety profile in this study of Chinese subjects with moderate‐to‐severe melasma.

## 1 Introduction

Melasma is a common acquired pigmentation disorder characterized by symmetrical brown macules on the face [1]. The exact pathogenesis of melasma remains unclear; however, several factors, including exposure to ultraviolet (UV) radiation, genetic predisposition, oral contraceptives, pregnancy, and female sex, have been found to increase the risk of melasma [2]. It can be triggered by UV radiation through the upregulation of melanocyte‐stimulating hormone (MSH) receptors [3], which facilitates recurrence. The prevalence of melasma is higher among more pigmented skin types, such as East Asians (Japanese, Koreans, and Chinese), Indians, Pakistanis, Middle Easterners, and Mediterranean‐African [4]. The prevalence was reported to be 8.8% [5] in premenopausal Latin women and 40% in South Asian women [6].

Melasma often develops over a long period of time and can significantly influence the quality of life [7]. Despite there being many treatment options available, the effective treatment of melasma remains difficult. Hydroquinone is considered the gold standard for topical treatment [8]; it was proven to be efficacious when compared to 1% flutamide cream [9]. In addition, cysteamine has been shown to be efficacious in melasma treatment. In a randomized, double‐blind clinical trial, the cysteamine group had significantly lower Melasma Area and Severity Index (MASI) scores than the placebo group over a 4‐month period (*p* = 0.04) [10].

Kligman and Willis first proposed the combination of 0.1% tretinoin, 5% hydroquinone, and 0.1% dexamethasone, known as the “Kligman formula”, for effectively treating melasma [11]. Furthermore, a triple combination cream (TCC) with 0.05% tretinoin, 4% hydroquinone, and 0.01% fluocinolone acetonide was developed by Galderma Laboratories and approved by the United States Food and Drugs Administration (FDA) in 2002 [12]. The brand name of this formulation, TRI‐LUMA, has been widely tested and used in Caucasian populations; however, no clinical trial based on the guidance of the FDA has been conducted in the Chinese population. Therefore, the efficacy and safety of TRI‐LUMA in the Chinese population remain unclear.

A multicenter, randomized, double‐blind, three‐arm, parallel‐group, placebo‐controlled clinical trial was conducted to determine whether the generic TCC is efficacious and safe in the Chinese population and to provide a reference for patients and health professionals. This report collects and discusses data from our hospital, which is an important center for this study.

The primary objective of this study was to evaluate the efficacy and safety of the generic TCC for the treatment of moderate‐to‐severe melasma. The secondary objective was to evaluate the clinical equivalence of the generic TCC and TRI‐LUMA in the treatment of moderate‐to‐severe facial melasma.

## 2 Materials and Methods

### 2.1 Study Design

The study procedures were performed in accordance with good clinical practice and local regulations. The Chinese National Medical Products Administration (NMPA) approved the study protocol before commencement. Furthermore, the study protocol was approved in September 2020 by the Ethics Committee of the Fourth Affiliated Hospital of Zhejiang University School of Medicine (S2020005) and was registered with the Chinese Clinical Trial Registry (ChiCTR2100043798). Informed consent was obtained from all patients before screening, and the process complied with ethical standards. This randomized, double‐blind, three‐arm, parallel‐group, placebo‐controlled clinical trial was performed at the Department of Dermatology of our hospital from October 2020 to July 2021.

### 2.2 Subjects

According to the inclusion and exclusion criteria, 53 subjects were recruited and randomized in this trial. The generic TCC, TRI‐LUMA, and placebo groups were allocated to patients at a ratio of 2:2:1.

This study included patients aged 18–60 years with moderate‐to‐severe facial melasma (severity score of 2 or 3 on a 0–3 scale, with 3 indicating severe severity) that was stable for at least 3 months at baseline. Patients were required to use the provided products, including facial cleansers, moisturizers, and sunscreens, and no depression or atrophy was observed in the affected areas.

Exclusion criteria included pregnancy or breastfeeding, facial skin conditions that could interfere with melasma diagnosis or assessment (e.g., acne, eczema, allergic dermatitis, scars, or dyschromic diseases, such as vitiligo, nevus fuscoceruleus zygomaticus, and Riehl's melanosis), low immune function, and known allergies to investigational or skincare products.

Before screening, a washout period was required for various treatments: 4 weeks for topical/oral drugs for melasma (e.g., hydroquinone, corticosteroids, retinoids) and for pigment‐affecting drugs (e.g., tryptophan, cysteine, tranexamic acid, reduced glutathione), as well as for bleaching products and UV light therapy. A 3‐month washout was required for acitretin, azathioprine, cyclosporine, interferon, isotretinoin, methotrexate, mycophenolate mofetil, sirolimus, and tacrolimus. Dermabrasion, peels, or laser treatments required a 6‐month washout, and anti‐inflammatory, antihistamine, or hypnotic treatments required a 2‐week washout.

A Wood lamp was used for fluorescence diagnosis to identify the type of melasma in each patient. Pigmented areas that appear enhanced under a Wood lamp indicate increased epidermal melanin (epidermal type), whereas nonenhanced areas suggest increased dermal melanin (dermal type). Areas showing both enhanced and nonenhanced characteristics indicate a mixed subtype of melasma (mixed type).

### 2.3 Intervention

The subjects were randomized to receive the generic TCC, TRI‐LUMA, or placebo, which was provided by Zhejiang Fonow Medicine Co. Ltd. A list of the randomized numbers and the corresponding treatment groups was generated using SAS, and each randomized number was assigned using the central randomization system (DAS for IWRS). A thin film of cream was applied to the affected area 30 min before bedtime for 8 weeks. During the day, subjects used a moisturizer to moisten the affected area and applied sunscreen to avoid exposure to sunlight.

### 2.4 Data Collection

An electronic case report form (e‐CRF) designed and implemented by DAS was used to collect the study data.

### 2.5 Efficacy Assessment

The subjects visited the investigator's center four times for assessments at baseline, week 1 (safety and compliance assessments only), week 4, and week 8.

The primary efficacy variable was the Melasma Severity Scale (MSS) score at week 8. The percentage of patients that achieved a reduction in the MSS score by 2 or 3 was compared between groups (effective: MSS score decline of 2 or 3; noneffective: MSS score decline < 2) [13]. The MSS was assessed with reference to a previous study [14].

The secondary efficacy variable was the percentage reduction in the MASI scores at weeks 4 and 8. The MASI, developed by Kimbrough‐Green et al. [15], was assessed at baseline, week 4, and week 8 by the same dermatologist, who was blinded to the treatment. A percentage reduction in the MASI score ≥ 80%, ≥ 50% to < 80%, ≥ 30% to < 50%, and < 30% indicated almost clear, marked improvement, minimal or moderate improvement, and stable or worsening, respectively.

The percentage reduction in the MASI score was calculated as follows: (MASI score at baseline—MASI score after treatment)/MASI score at baseline × 100%.

The effective rate was calculated as follows: (number of almost clear cases + number of marked improvements)/total number of cases × 100.

### 2.6 Safety Assessments

Treatment‐emergent adverse events (TEAEs) were categorized according to the Common Terminology Criteria for Adverse Events (CTCAE) version 5.0. Epidermal atrophy and telangiectasia were monitored as hormone‐related adverse events.

### 2.7 Statistical Analyses

The efficacy variable was analyzed using the modified intention‐to‐treat (ITTm) principle. We analyzed all participants who were randomly assigned to receive topical treatment at least once. Missing data were imputed using the last observation carried forward (LOCF) method.

The efficacy rates between the study groups were compared using Fisher's exact test. Demographic factors were compared among the three groups using the Kruskal–Wallis test and one‐way analysis of variance (ANOVA). An ANCOVA model was used to compare the percentage reduction in the MASI score from baseline to weeks 4 and 8 between the study groups. The Chi‐square test was used to compare the incidence of specific symptoms (erythema, irritation, and skin exfoliation) among the groups. Other adverse events, such as epidermal atrophy, telangiectasia, and hyperpigmentation, were recorded in every group. Data were analyzed using IBM SPSS version 27, and significance was set at *p* < 0.05.

## 3 Results

### 3.1 Demography

**FIGURE 1:**
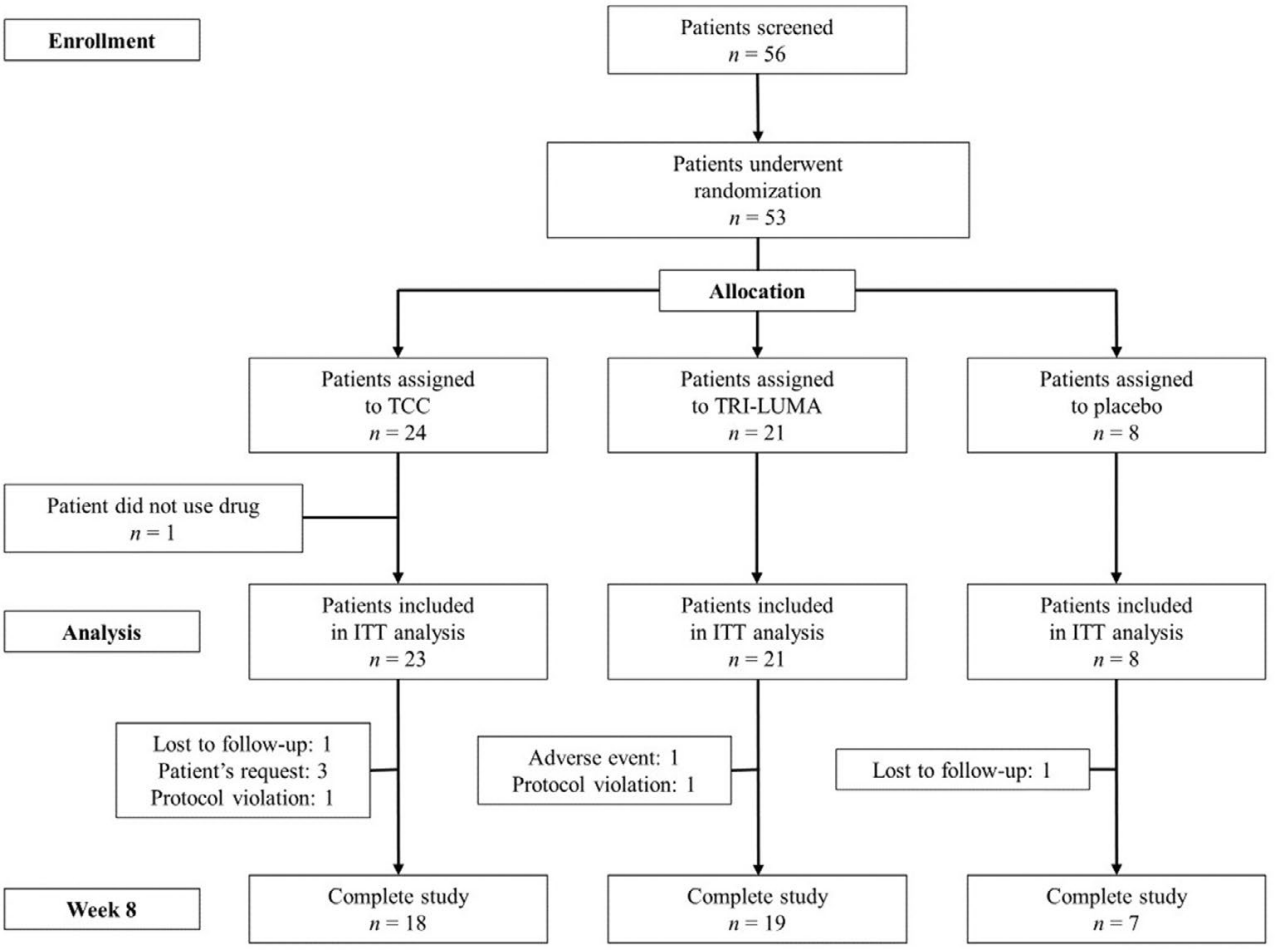
Flow diagram of participants in the study. ITT, intention‐to‐treat.

A flow diagram of the participants is shown in Figure 1. A total of 56 patients were screened, and 53 were randomized. As one patient randomized to the TCC group did not use the drug, 52 patients were analyzed (23 in the TCC group, 21 in the TRI‐LUMA group, and eight in the placebo group). Overall, 44/53 patients (83.0%) completed the study: 18 (75%) in the TCC group, 19 (90.5%) in the TRI‐LUMA group, and seven (87.5%) in the placebo group. The patient demographic characteristics are summarized in Table 1. The mean patient age was 44.3 years in the TCC group, 46.0 years in the TRI‐LUMA group, and 41.3 years in the placebo group. Most subjects were female, and most had epidermal melasma, which was identified using a Wood lamp.

**TABLE 1:** Patient demographics and melasma characteristics at baseline.

	TCC (*n* = 24)	TRI‐LUMA (*n* = 21)	Placebo (*n* = 8)	Total (*n* = 53)	*p*
Gender, *n* (%)					0.248
Male	3 (12.5)	0 (0)	1 (12.5)	4 (7.5)	
Female	21 (87.5)	21 (100)	7 (87.5)	49 (92.5)	
Age (years)					0.062
Mean ± SD	44.3 ± 4.1	46.0 ± 5.4	41.3 ± 4.5	44.5 ± 4.9	
Median	43	46	43.5	44	
Range	38–55	36–55	33–45	33–55	
Race, *n* (%)					/
Asian	24 (100)	21 (100)	8 (100)	53 (100)	
Type of melasma, *n* (%)					0.299
Epidermal	16 (66.7)	18 (85.7)	6 (75)	40 (75.5)	
Dermal	1 (4.2)	1 (4.8)	1 (12.5)	3 (5.7)	
Mixed	7 (29.2)	2 (9.5)	1 (12.5)	10 (18.9)	
MSS score					0.293
Moderate (2), *n* (%)	7 (29.2)	11 (52.4)	3 (37.5)	21 (39.6)	
Severe (3), *n* (%)	17 (70.8)	10 (47.6)	5 (62.5)	32 (60.4)	
Mean ± SD	2.7 ± 0.5	2.5 ± 0.5	2.6 ± 0.5	2.6 ± 0.5	
MASI score					0.758
Mean ± SD	21.4 ± 9.4	19.9 ± 7.2	19.2 ± 8.0	20.4 ± 8.3	

Abbreviations: MASI, Melasma Area and Severity Index; MSS, Melasma Severity Scale; TCC, triple combination cream.

### 3.2 Efficacy Results

**FIGURE 2:**
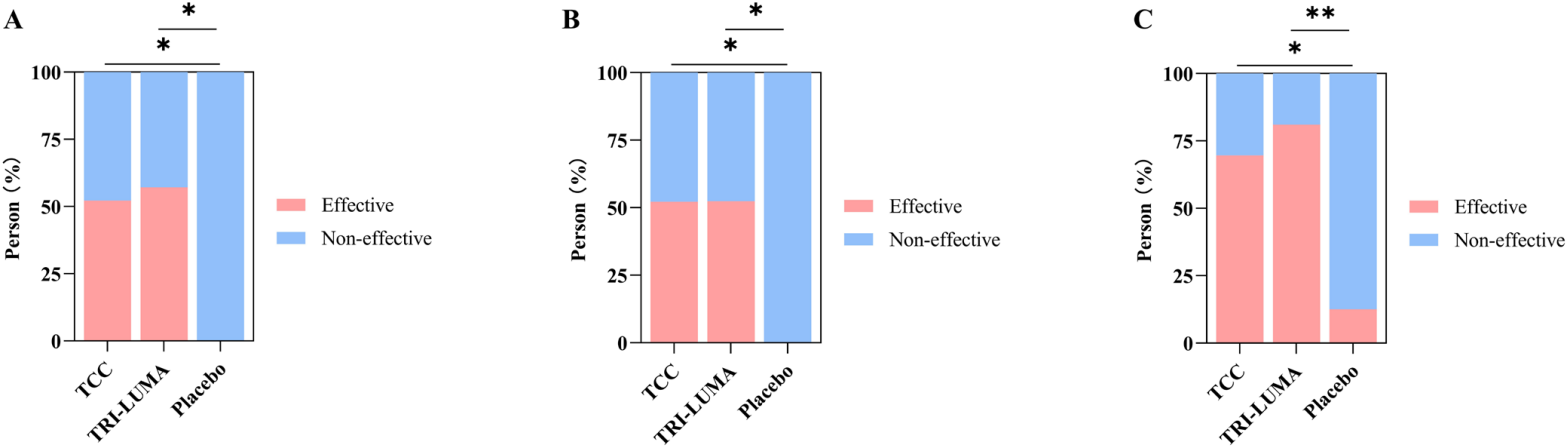
MSS and MASI evaluation after treatment in the ITT population. The distribution of patients who achieved effective treatment^a^ in the three groups according to the MSS score at week 8 (A). The distribution of patients who achieved effective treatment^b^ in the three groups based on the percentage reduction in the MASI score at week 4 (B) and week 8 (C). ^a^A reduction of 2 or 3 in the MSS score. ^b^A percentage reduction in the MASI score ≥ 80%, and ≥ 50% to < 80%. *p < 0.05, **p < 0.01. MASI, Melasma Area and Severity Index; MSS, Melasma Severity Scale; ITT, intention‐to‐treat.

Figure 2 shows the distribution of patients who achieved effective treatment in the three groups according to the MSS and MASI evaluation. Using the decline in the MSS score by 2 and 3 at week 8, 12/23 patients (52.2%) in the TCC group achieved effective treatment, compared with 12/21 patients (57.1%) in the TRI‐LUMA group (*p* > 0.05), and 0/8 patients (0%) in the placebo group in the ITT‐LOCF population (*p* < 0.05) (Figure 2A). At weeks 4 and 8, the percentage reduction in the MASI score in both the TCC and TRI‐LUMA groups was statistically superior to that in the placebo group (*p <* 0.05), with no significant difference between the TCC and TRI‐LUMA groups. The effective rate was 52.2% vs. 52.4% (*p* > 0.05) at week 4 and 69.6% vs. 81.0% (*p >* 0.05) at week 8 (Figure 2B,C).

**FIGURE 3:**
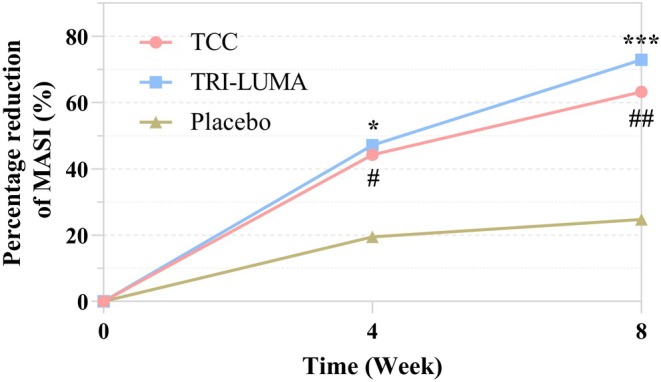
Evolution of the percentage reduction in the MASI score of each treatment group. # The improvement between the TCC and placebo group is significant (*p* < 0.05), ##*p <* 0.01. *The improvement between the TRI‐LUMA and placebo group is significant (*p <* 0.05), ***p < 0.001. MASI, Melasma Area and Severity Index.

Figure 3 illustrates the evolution of the percentage reduction in MASI scores between groups at weeks 4 and 8. The mean percentage reduction in the MASI score at week 4 was 44.2% ± 33.9% in the TCC group, 47.1% ± 27.1% in the TRI‐LUMA group (*p >* 0.05, vs. TCC), and 19.5% ± 21.2% in the placebo group (*p <* 0.05, vs. TCC). The mean percentage reduction in the MASI score at week 8 was 63.2% ± 36.2% in the TCC group, 72.9% ± 27.0% in the TRI‐LUMA group (*p >* 0.05, vs. TCC), and 24.7% ± 31.1% in the placebo group (*p <* 0.01, vs. TCC).

**FIGURE 4:**
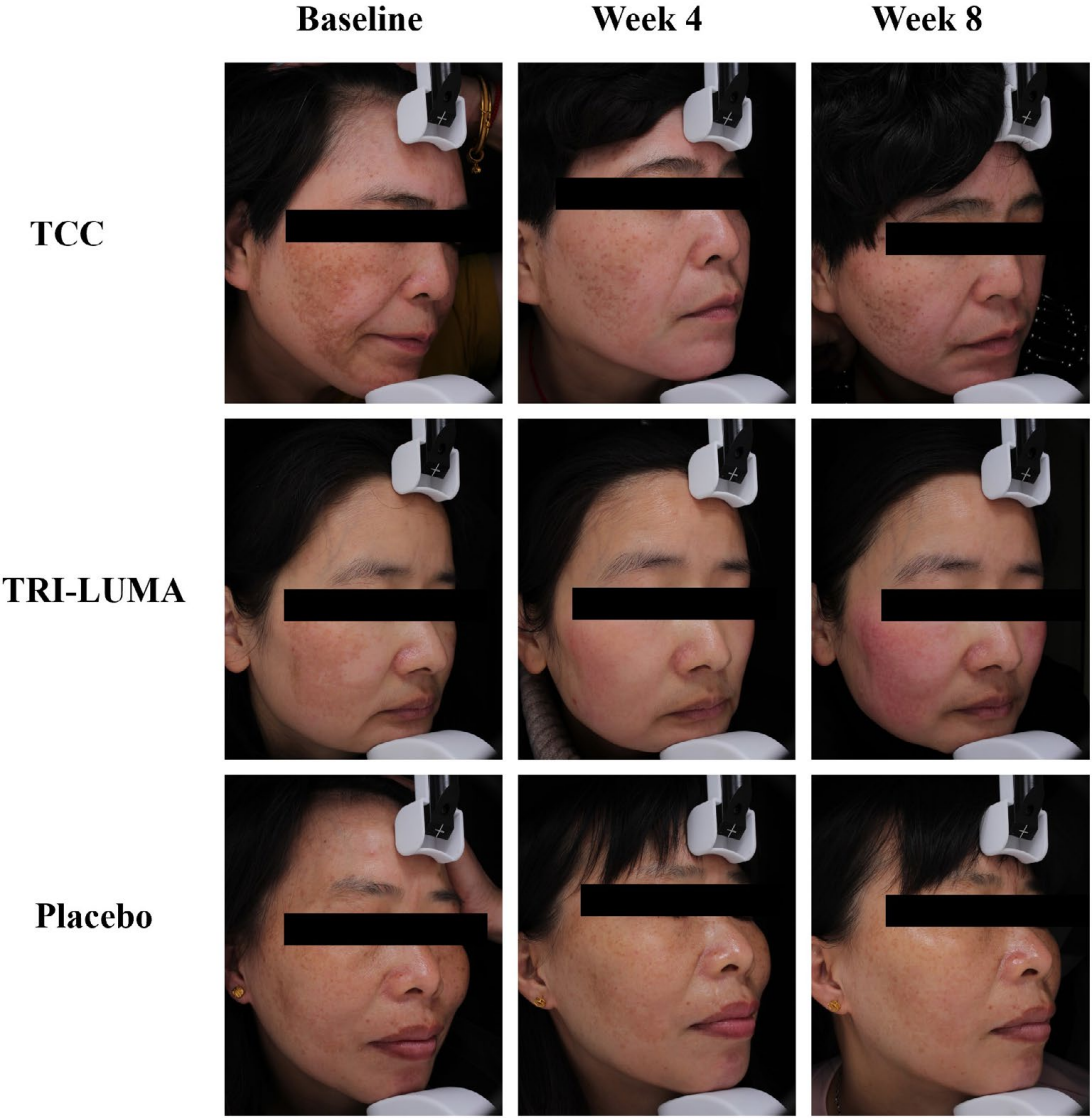
Clinical appearance of patients treated with TCC, TRI‐LUMA, and placebo at baseline, week 4, and week 8.

Figure 4 shows photographs of the patients after 8 weeks of treatment with TCC, TRI‐LUMA, and placebo. After 8 weeks of treatment, patients in the TCC and TRI‐LUMA groups achieved a greater reduction in the color and area of melasma.

### 3.3 Safety Results

**FIGURE 5:**
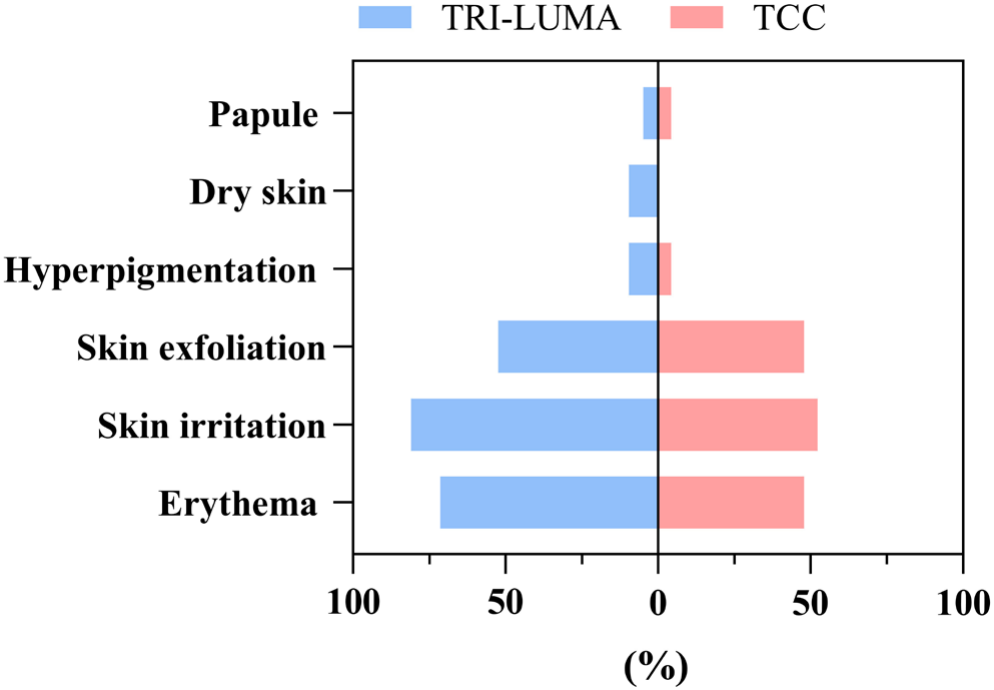
Types of TEAEs and number of cases in the TCC and TRI‐LUMA groups. TEAE, treatment‐emergent adverse event.

In the TCC group, 69.6% (16/23) of patients and in the TRI‐LUMA group, 90.5% (19/21) of patients experienced TEAEs (*p* > 0.05). No TEAEs were observed in the placebo group. Figure 5 shows that the most common TEAEs were erythema (11/23 patients, 47.8% for TCC vs. 15/21 patients, 71.4% for TRI‐LUMA, *p* > 0.05), skin irritation (12/23 patients, 52.2%, vs. 17/21 patients, 81.0%, *p* < 0.05), and skin exfoliation (11/23 patients, 47.8%, vs. 11/21 patients, 52.4%, *p* > 0.05). Other TEAEs included hyperpigmentation (one patient with TCC vs. two patients with TRI‐LUMA), dry skin (zero vs. two patients), and papules (one vs. one patient). Four TEAEs (one case each of erythema, skin irritation, skin exfoliation, and hyperpigmentation) were grade 2 in two patients from the TCC group. The other TEAEs in the TCC and TRI‐LUMA groups were all grade 1. One patient in the TRI‐LUMA group withdrew from the trial because of skin exfoliation. No patients in the TCC group withdrew from the trial because of TEAEs. Telangiectasia and skin atrophy were not reported after 8 weeks of treatment.

## 4 Discussion

Melasma is an acquired pigmentation disorder characterized by asymptomatic, symmetrical pigmented macules on the face [16]. Various factors can increase the risk of melasma, which can occur in all populations. The prevalence ranges widely at 1%–50%, especially in females and darker skin types [17].

Although hydroquinone cream has been approved for the treatment of melasma, it is associated with a high incidence of adverse effects. To alleviate the melanocyte‐derived cytotoxicity of hydroquinone [18], a new combination of hydroquinone, tretinoin, and corticosteroids, known as TRI‐LUMA, has been developed. Tretinoin can increase the smoothness of the skin through epidermal hyperplasia, compaction of the stratum corneum, and several other mechanisms that can accelerate depigmentation and mitigate the side effects of hydroquinone [19]. Fluocinolone acetonide is a synthetic corticosteroid that can inhibit IL‐2 mediated T cell proliferation and cytokine production and simultaneously induce the expression of the pro‐survival receptor IL‐7R [20]; this ingredient could decrease the incidence of adverse events. The first clinical trial of this formula was a multicenter, open‐label, 8‐week study with extended treatment for up to 12 months, which indicated that this cream was well tolerated [21].

TRI‐LUMA is a common method for the treatment of melasma in the United States and is considered the gold standard treatment for melasma and the first‐line medication in clinical practice. However, previous studies have primarily focused on Caucasian populations. Drugs may have different pharmacokinetics and functions in different races, and the same disease may have different epidemiological and pathological manifestations in different continents and populations. Specifically, the incidence of melasma is higher in the Chinese population than in the Caucasian population. Therefore, this treatment may have different efficacy and safety profiles in Chinese patients. This is the first clinical trial of the generic TCC in Chinese subjects based on the FDA requirements of an equivalence study. According to the FDA‐approved label of TRI‐LUMA cream, 38% of melasma patients achieved a 2‐ or 3‐grade reduction in the MSS score after 8 weeks of treatment. Detailed in the draft guidance on fluocinolone acetonide, hydroquinone, and tretinoin (FDA, 2015) [13], the equivalent cut‐off value was taken as ±20%, the one‐sided *α* = 0.05, the *β* = 0.2, and the dropout rate was 20%. The sample size was calculated using PASS14.0 software. If the generic TCC, TRI‐LUMA, and placebo ratios were 2:2:1, the sample sizes of the three groups would be 128, 128, and 64, respectively. This national multicenter trial enrolled 320 patients, which allowed adequate performance of statistical analyses; however, the data has not yet been analyzed. This article is a single‐center data analysis of the aforementioned multicenter trial with a smaller sample size; therefore, Fisher's exact test was used for efficacy analysis.

In this study, the definition of effective treatment was a decline in the MSS score ≥ 2. According to this standard, the effective rates of the generic TCC and TRI‐LUMA were > 50%, with no significant difference between these two groups; however, both groups showed prominent efficacy compared with the placebo group. This study demonstrates that the generic TCC has comparable efficacy to TRI‐LUMA at week 8. According to previous studies, the treatment success rate for TRI‐LUMA in Asian populations with moderate‐to‐severe melasma was 64.2% [22]. Treatment success is defined as an MSS score of 0 or 1 at week 8 of treatment. Therefore, patients with an MSS score decline of 1 were included, which resulted in the increased rate. In another 8‐week clinical trial conducted by Taylor in 2003, the rate of patients whose MSS score achieved 0 or 1 at week 8 was 77% [14]. Most patients enrolled in the Taylor study were non‐Asians. Kang et al. [23] conducted a histopathological examination and ultra‐micropathological study on 56 Korean patients with facial melasma and found that melanocytes in the melasma lesion area were significantly increased compared to those in the non‐lesion area. Furthermore, the number of melanocytes in the lesion area increased in 84% of melasma patients. In a study of 21 non‐Asian patients with melasma by Grimes et al. [24], the pigmentation of melasma lesions increased without a change in the number of melanocytes; the increase in pigmentation was mainly due to an increase in melanin synthesis and transport. There are few comparative studies on the histopathology of melasma lesions and non‐lesions, with inconsistent results. Therefore, it remains uncertain whether the difficulty in treating melasma in Asian populations is related to an increased number of melanocytes in the lesions.

In this study, the evaluation of the MASI scores presented results similar to those of the MSS scores regarding the efficacy differences between the treatment groups. At weeks 4 and 8, the definition of effective treatment was a ≥ 50% reduction in the MASI score, with no significant difference between the generic TCC and TRI‐LUMA groups at either time point. Both showed a higher efficacy rate than the placebo group, indicating that the generic TCC had the same onset as TRI‐LUMA. Although there was no significant difference between these two groups at week 8 according to the MASI score, it seems that TRI‐LUMA had a higher efficacy rate than the generic TCC (81.0% vs. 69.6%); however, this may be because the sample size does not meet the power of a test. Therefore, in clinical practice, physicians and patients must refer to data from the entire clinical trial. The efficacy of TRI‐LUMA was 59.5%, whereas that of the generic TCC was 61.6%, at week 8 in the national multicenter clinical trial, with no statistical significance between these groups, corresponding to the present study results. At weeks 4 and 8, the percentage reduction in the MASI score in both the generic and TRI‐LUMA groups was significantly higher than that in the placebo group. There was no significant difference between the two positive drug groups, indicating that the generic TCC had a rapid onset of response in the treatment of melasma and continued to maintain efficacy at week 8, which was almost the same as for TRI‐LUMA. The reduction in the MASI score after treatment was similar to that reported by Chan et al. [22]. Since the generic TCC has equal efficacy to TRI‐LUMA, it could be seen as a good choice for economic benefits.

The incidence of TEAEs was lower in the generic TCC group than that in the TRI‐LUMA group (69.6% vs. 90.5%); however, the difference was not statistically significant. No TEAEs were observed in the placebo group. The most frequent TEAEs were erythema, irritation, and skin exfoliation, which are predictable adverse reactions to hydroquinone and tretinoin. The incidence of irritation in the generic TCC group was significantly lower than in the TRI‐LUMA group (52.3% vs. 81%, respectively), suggesting that the generic TCC has a significant advantage in reducing irritation. Regarding the severity of TEAEs, two subjects in the generic TCC group had grade 2 TEAEs; none of them quit the study because of TEAEs. The remaining TEAEs were of grade 1. Only one patient treated with TRI‐LUMA withdrew from this study because of skin exfoliation, confirming that the generic TCC and TRI‐LUMA groups had the same safety profiles. Clinicians are concerned about the risk of telangiectasia and skin atrophy when treating melasma with topical corticosteroids. Kligman et al. [11] suggested combining corticosteroids with retinoids to prevent steroid‐induced skin atrophy. Furthermore, the fluocinolone acetonide used in this study is a low‐potency, fluorinated corticosteroid. Telangiectasia and skin atrophy were not observed in any participants at week 8. Furthermore, hydroquinone can induce exogenous ochronosis, which is characterized by reticulated hyperpigmentation of the face [25]. Hyperpigmentation was observed in both groups treated with hydroquinone; however, other factors also influence pigmentation disorders, such as sun exposure and sleep deprivation. Thus, the cause of hyperpigmentation is uncertain.

The study showing that generic TCC is as effective and safe as TRI‐LUMA for treating melasma could impact clinical practice in China. Being more affordable, generic TCC might be used more frequently, making treatment more accessible without sacrificing quality. Additionally, the availability of locally produced options could benefit patients with limited financial resources or those in remote areas. To address long‐term effects and adherence challenges with hydroquinone‐based treatments like TRI‐LUMA and generic TCC, further studies on long‐term safety are crucial. These treatments, effective for melasma, may cause side effects such as exogenous ochronosis and skin atrophy with prolonged use. Although no such effects were observed by week 8, comprehensive patient education on managing side effects and ensuring treatment accessibility and affordability is essential to encourage consistent use.

The main limitations of this study are the small sample size and short study duration. Larger multicenter studies with a power analysis or confidence intervals for equivalence are needed to further address potential differences in efficacy and safety between generic TCC and TRI‐LUMA. Due to the limitations of the small sample size, no subgroup analyses were performed. Data collected from the national multicenter trial can be considered for subgroup analysis, and extension trials could be conducted to explore recurrence, demonstrate the safety and tolerance of TCC with long‐term use, and determine the therapy duration.

## 5 Conclusion

In this study, fluocinolone acetonide, hydroquinone, and tretinoin cream showed satisfactory efficacy and safety in Chinese patients with moderate‐to‐severe melasma compared to placebo, and the depigmentation effect of TCC was proven to be reliable in this race. Additionally, the generic TCC has an efficacy rate equal to that of TRI‐LUMA, which provides another choice for patients and health professionals when facing melasma.

## Author Contributions

Honghua Hu drafted the manuscript and Lunfei Liu revised the paper. Pengfei Zhou, Hongliang Yao, Chengyao Zhu, Bo Shen, Bo Feng, and Xiangke Yu acquired and analyzed the data.

## Ethics Statement

The authors confirm that the ethical policies of the journal, as noted on the journal's author guidelines page, have been adhered to. The study protocol was approved in September 2020 by the Ethics Committee of the Fourth Affiliated Hospital of Zhejiang University School of Medicine (ZJU4H) (S2020005) and was registered with the Chinese Clinical Trial Registry under the identifier ChiCTR2100043798. Informed consent was obtained from all patients before screen, and the process was in compliance with ethical standards.

## Consent

The photographs used in this manuscript were original and collected during the trial, subjects had agreed and authorized us to use these pictures in public articles. These pictures had been processed to protect privacy.

## Conflicts of Interest

The authors declare no conflicts of interest.

## Data Availability

Research data are not shared.
